# Ultrasound-Guided Vacuum-Assisted Excision (VAE) in Breast Lesion Management: An Experimental Comparative Study of Two Different VAE Devices Across Various Aspiration Levels and Window Sizes

**DOI:** 10.3390/diagnostics15030272

**Published:** 2025-01-24

**Authors:** Serena Carriero, Maurizio Cè, Matilde Pavan, Mariassunta Roberta Pannarale, Giulia Quercioli, Sveva Mortellaro, Alessandro Liguori, Maria Cosentino, Maria Iodice, Marta Montesano, Giulia Querques, Carolina Lanza, Salvatore Alessio Angileri, Pierpaolo Biondetti, Filippo Pesapane, Gianpaolo Carrafiello, Sonia Santicchia

**Affiliations:** 1 Radiology Department, Fondazione IRCCS Cà Granda Ospedale Maggiore Policlinico, Via Francesco Sforza 35, 20122 Milan, Italy; 2 Postgraduate School in Radiodiagnostics, Università degli Studi di Milano, Via Festa del Perdono 7, 20122 Milan, Italy; 3 Breast Imaging Division, Radiology Department, IEO European Institute of Oncology IRCCS, 20141 Milan, Italy; 4 Department of Oncology and Hematology-Oncology, Università degli Studi di Milano, Via Festa del Perdono 7, 20122 Milan, Italy

**Keywords:** breast, sample volume, vacuum-assisted biopsy, vacuum-assisted excision, breast cancer, breast benign lesions, B3

## Abstract

**Background/Objectives**: Vacuum-assisted excision (VAE) is a minimally invasive technique for breast tumor treatment, offering precision, comfort, and quick recovery. It is widely used for benign breast lesions and is playing an increasingly important role in the therapeutic management of non-surgical patients or patients who refuse surgery. Optimal outcomes require an understanding of device features to tailor treatment to each lesion. The Mammotome^®^ Elite 10G operates in a fixed mode, while the Mammotome^®^ Revolve EX 8G offers multiple aspiration levels and aperture windows for greater versatility. This study analyzed the specimen features (weight and length), comparing the weight obtained from two different VAE systems to aid the appropriate selection of a device based on the clinical setting. It also determined the number of specimens needed to achieve the 4 g diagnostic threshold. **Methods**: The Mammotome^®^ Elite 10G and the Mammotome^®^ Revolve EX were evaluated under controlled conditions. For Mammotome^®^ Revolve EX, combinations of five aspiration levels and three aperture lengths (12 mm, 18 mm, and 25 mm) were tested. Twelve samples were collected from a chicken breast phantom for each setting. Specimen weights and the minimum excisions required to reach the 4 g threshold were analyzed. **Results**: The mean weight per sample for the Mammotome^®^ Elite 10G was 0.16 ± 0.04 g. For the Mammotome^®^ Revolve EX, the weights increased with aperture size and aspiration level, ranging from a minimum of 0.132 ± 0.028 g (a window length of 12 mm and aspiration level 1) to a maximum of 0.407 ± 0.055 g (a window length of 25 mm and aspiration level 5). The 25 mm window at aspiration level 5 achieved the 4 g threshold in as few as 10 samples. By comparison, the Mammotome^®^ Elite required up to 26 samples. **Conclusions**: Compared to the Mammotome Elite, Mammotome^®^ Revolve EX offers superior versatility and efficiency, reducing patient discomfort by minimizing the required samples. Its technical advantages make it a valuable tool for both diagnostic and therapeutic applications.

## 1. Introduction

Vacuum-assisted excision (VAE) is a non-surgical technique derived from vacuum-assisted breast biopsy (VABB), a diagnostic procedure well-known for breast lesions [1]. The main characteristic of VAE is that it employs a large needle, typically up to 8G, allowing multiple cores to be obtained while the needle remains in place after a single insertion, achieving entire lesion removal under Ultrasound (US) [2].

VAE has been shown to possess excellent excisional power, as it is capable of achieving a tissue sample volume or weight comparable to that of a diagnostic surgical excision, approximately 4 g [3]. This sample can be obtained using VAE by varying the number of specimens obtained, with the total volume depending on the needle size used [4].

Due to its excision ability, several studies have investigated the possible therapeutic role of VAE, and since receiving approval from the Food and Drug Administration (FDA) in 2002, VAE has become a well-established therapeutic alternative for managing benign lesions nowadays. Numerous studies have demonstrated that VAE is a safe and effective technique, offering high rates of complete resection and low rates of recurrence and complications [5]. Following the procedure, no ultrasonographically detectable evidence of the original lesion is observed in up to 98% of patients [6].

However, for the management of B3 lesions or high-risk lesions, there is more controversy about whether they can be treated with VAE instead of surgery. International Consensus Conferences [7,8] and, more recently, European guidelines [9] support minimally invasive management of B3 lesions using therapeutic VAE after a thorough evaluation by a multidisciplinary team considering lesion size, presence of atypia, and imaging discordance. In particular, one of the most critical factors influencing the success of VAE in these cases is lesion size, with lesions smaller than 1.5 cm more likely to undergo complete resection with vacuum-assisted excision [10].

On the other hand, the role of VAE in breast cancer management remains an area with many uncertainties, as there is currently no prospective randomized evidence to support its routine use. To date, only a limited number of studies have demonstrated that small, low- to intermediate-grade pT1a/b breast tumors can potentially be completely resected percutaneously with VAE [11,12]. This can have a big impact on the management of the breast cancer patient’s treatment since the introduction of screening mammography has led to an increase in breast cancer incidence and a notable rise in the detection of smaller tumors (<2 cm) [13]. Prospective trials, such as the ongoing SMALL [14] and MINIVAB [15] studies, are, therefore, essential to provide robust evidence and support a shift toward minimally invasive techniques for the management of breast cancer.

To embrace the future of vacuum-assisted excision for small tumors, it is essential to understand the technical capabilities of the available devices, optimizing the technologies we have in relation to the diverse clinical scenarios that may arise.

Moreover, it is necessary to identify which of the available devices have greater excisional power and the most suitable technical characteristics in relation to the size and location of the lesion.

Nowadays, there is a wide range of devices available, and we focused our attention on the comparison of the Mammotome^®^ Elite (Devicor Medical Products, Inc., Cincinnati, OH, USA) and the newest Mammotome^®^ Revolve EX (Devicor Medical Products, Inc., Cincinnati, OH, USA). The Mammotome^®^ Elite is a tetherless device widely utilized in clinical settings due to its high accuracy, safety, and cost-effectiveness, as well as its low rates of pathological underestimation and hematoma formation [16,17].

The Mammotome^®^ Revolve EX device is a newer system designed for lesion excision with continuous sampling capabilities, five different levels of aspiration, and three different aperture lengths (Devicor Medical Products, Inc., Mammotome RevolveTM EX, DUAL VACUUM-ASSISTED BREAST BIOPSY SYSTEM. https://www.mammotome.com/uk/en/products/revolve-ex, accessed on 5 December 2024).

The growing adoption of minimally invasive options for breast tumor removal has prompted us to evaluate the tissue extraction capabilities of the Mammotome^®^ Revolve EX compared to the Mammotome^®^ Elite, considering all available settings and including all the different aspiration levels and window sizes.

## 2. Materials and Methods

We evaluated the excision performance of two commercially available VAE systems under controlled conditions: Mammotome^®^ Elite 10G (Devicor Medical Products, Inc., Cincinnati, OH, USA) and Mammotome^®^ Revolve EX (Devicor Medical Products, Inc., Cincinnati, OH, USA). The Mammotome^®^ Elite is a tetherless device equipped with an integrated vacuum system that operates with a single level of aspiration. It is available with 13G or 10G caliber needles, featuring aperture lengths of 18.4 mm and 19.1 mm, respectively. In contrast, the Mammotome^®^ Revolve EX is a tethered system connected to a dual vacuum technology that offers five adjustable aspiration levels. This system utilizes an 8G caliber needle capable of continuous tissue acquisition and provides three aperture window sizes: 12 mm, 18 mm, and 25 mm.

### 2.1. Sample Preparation and Data Collection

**Figure 1:**
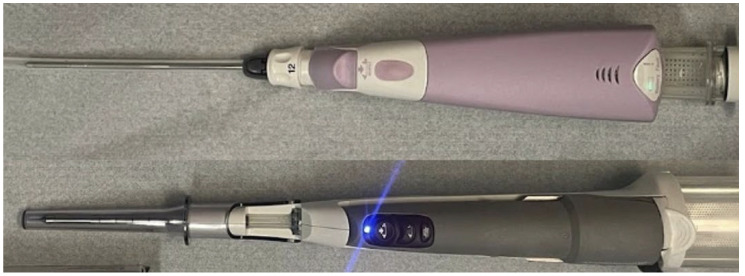
The two devices utilized in the study: at the top, the Mammotome^®^ Elite, and at the bottom, the Mammotome^®^ Revolve EX.

To minimize variability in the material, all samples were collected using a phantom prepared from chicken breasts purchased on the same day from the same supplier. Sampling was performed by a radiologist with five years of experience in interventional breast radiology. The operator carefully targeted different areas of each chicken breast to avoid overlapping sampling sites. The material used is illustrated in Figure 1.

Samples were collected using the Mammotome^®^ Elite 10G in its default configuration and the Mammotome^®^ Revolve EX across all combinations of its five aspiration levels and three window aperture lengths. To optimize resource utilization, three needles were used for all measurements. However, it is important to acknowledge that the needles may have experienced physical wear due to their repeated use, which may have influenced the quality of the measurements, representing a potential limitation of this study.

**Figure 2:**
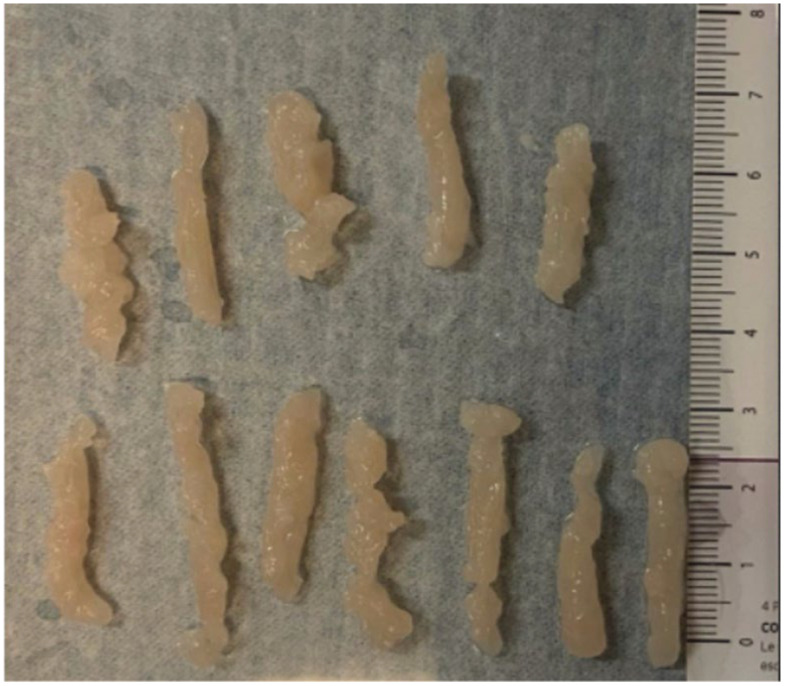
Phantom’s fragments obtained with Mammotome^®^ Revolve EX (aspiration level 5 and 18 mm window) laid out on a flat surface alongside a ruler for size reference.

**Figure 3:**
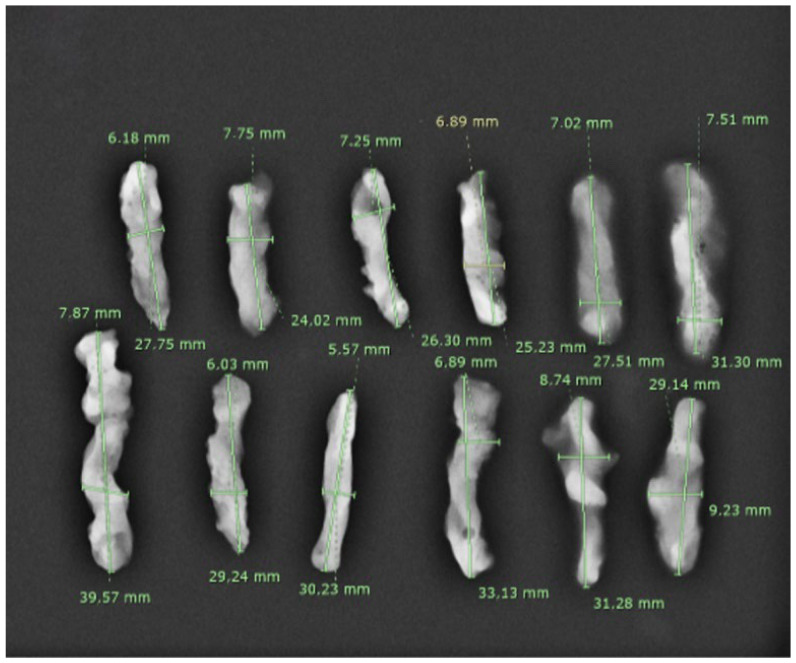
The X-ray scan of the fragments acquired using the Mammotome^®^ Revolve EX (aspiration level 5 and 25 mm window) with the measurements of each one.

**Figure 4:**
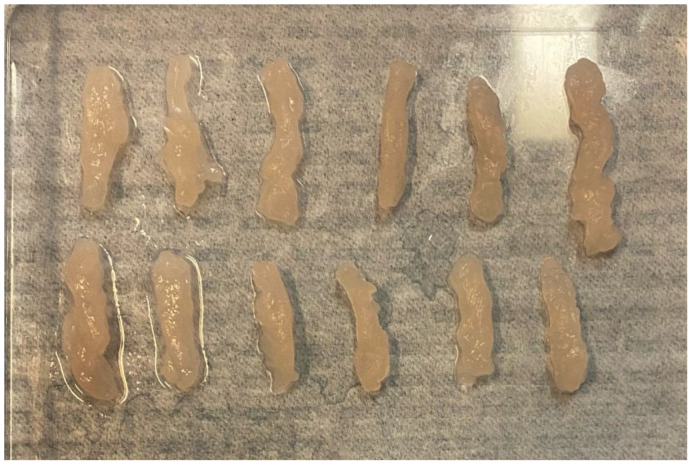
Fragments of the phantom obtained using the Mammotome^®^ Elite displayed on a flat surface.

The weight of the fragments was recorded using a high-resolution precision balance (KUBEI Bilance), which provides an accuracy of 1 mg. The fragments were carefully placed in dedicated containers and radiographed, and the resulting images were transmitted to the Picture Archiving and Communication System (PACS) for precise length measurement, ensuring greater reproducibility (Figure 2, Figure 3 and Figure 4).

All data were recorded in an Excel file (Microsoft, Redmond, WA, USA), with the variables coded as follows: R_WindowLength_AspirationLevel_W for the weight of the fragments and R_WindowLength_AspirationLevel_L for the length of the fragments for the Mammotome^®^ Revolve EX, and E_W for the weight of the fragments and E_L for the length of the fragments for the Mammotome^®^ Elite.

### 2.2. Data Analysis

The data analysis and graphical representation were performed using R software (Version 4.3.1., R Foundation for Statistical Computing, Vienna, Austria). The mean weight, standard deviation, mean length, and standard deviation for each combination of aspiration level and needle length were calculated. These results were visualized through boxplots and histograms grouped by needle length and aspiration pressure to facilitate interpretation.

In line with the approach outlined by Preibsch et al. [4], we estimated the minimum number of excisions required to reach a predefined weight threshold for the samples. A table was developed for each needle–aspiration system combination, detailing the estimated minimum number of fragments required to achieve the target weight of 4 g, which corresponds to the weight value of a diagnostic surgical excision, as recommended by UK guidelines for vacuum-assisted excision (VAE) procedures [3,8].

## 3. Results

Using the Mammotome^®^ Revolve EX, a total of 180 samples were collected across all combinations of needle window size and aspiration level, with 12 samples taken for each combination. Additionally, 12 samples were obtained using the Mammotome^®^ Elite, bringing the total number of samples to 192.

**Figure 5:**
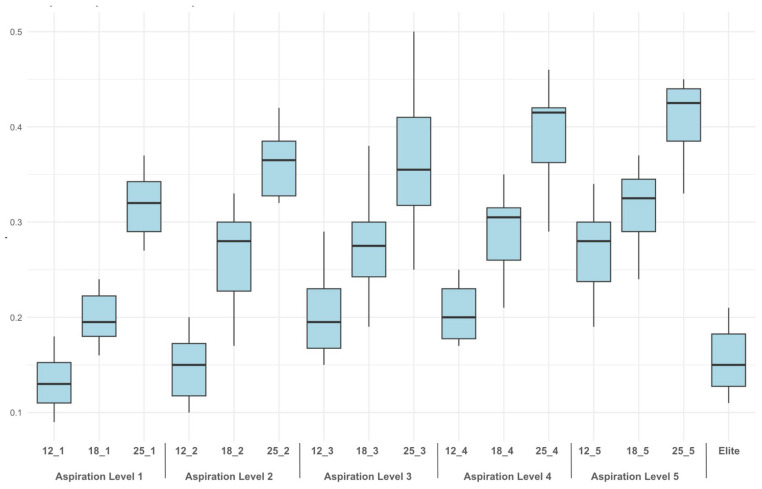
Boxplot showing the mean fragment weights (in grams) for the various combinations of Mammotome^®^ Revolve, expressed as WindowLength_AspirationLevel compared to Mammotome^®^ Elite (last column on the right).

The mean weights of the fragments and their variability across different combinations are depicted in the boxplot shown in Figure 5 and presented in Table 1.

**Table 1:** Table 1 presents the mean fragment weights for each combination of aspiration level and window length using the Mammotome^®^ Revolve. The data are grouped by window length, highlighting the variations in mean weights across different aspiration levels. The table includes the mean weight (g) and standard deviation (SD) for each combination; for a better visual assessment of the differences between combinations, the mean values are also presented as a histogram, grouped by aspiration levels (Figure 6).

Window.Length.mm	Aspiration.Level	Mean.Weight.g ± SD
12	1	0.132 ± 0.028
12	2	0.148 ± 0.034
12	3	0.204 ± 0.047
12	4	0.206 ± 0.033
12	5	0.268 ± 0.046
18	1	0.198 ± 0.026
18	2	0.264 ± 0.050
18	3	0.278 ± 0.057
18	4	0.293 ± 0.056
18	5	0.326 ± 0.053
25	1	0.319 ± 0.034
25	2	0.354 ± 0.052
25	3	0.360 ± 0.068
25	4	0.394 ± 0.054
25	5	0.407 ± 0.055

**Figure 6:**
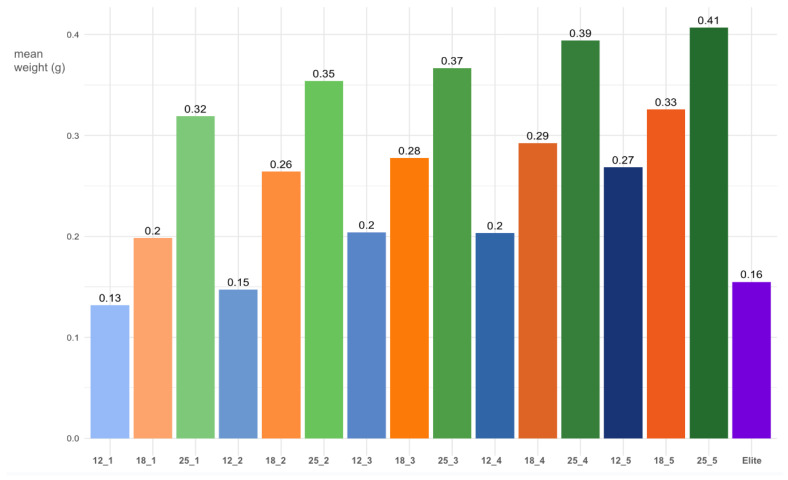
Mean weight (in grams) for the different combinations of Mammotome^®^ Revolve expressed as WindowLength_AspirationLevel. Blue bars represent the 12 mm window, orange bars the 18 mm window, and green bars the 25 mm window. Darker shades indicate higher aspiration levels. The purple bar represents the control with Mammotome^®^ Elite (window length: 19.1 mm).

Regarding fragments collected with the Mammotome^®^ Revolve EX, for the 12 mm window length, the mean fragment weight increased with aspiration level, starting at 0.132 ± 0.028 g at aspiration level 1, rising to 0.148 ± 0.034 g at level 2, 0.204 ± 0.047 g at level 3, 0.206 ± 0.033 g at level 4, and reaching 0.268 ± 0.046 g at level 5.

For the 18 mm window length, the mean fragment weight also showed an increasing trend, starting at 0.198 ± 0.026 g at aspiration level 1, increasing to 0.264 ± 0.050 g at level 2, 0.278 ± 0.057 g at level 3, 0.293 ± 0.056 g at level 4, and culminating at 0.326 ± 0.053 g at level 5. For the 25 mm window length, the mean fragment weight was consistently higher across all aspiration levels. It began at 0.319 ± 0.034 g at aspiration level 1, increased to 0.354 ± 0.052 g at level 2, 0.360 ± 0.068 g at level 3, 0.394 ± 0.054 g at level 4, and reached its maximum at 0.407 ± 0.055 g at level 5. Regarding fragments collected with the Mammotome^®^ Elite device, the mean fragment weight was 0.16 ± 0.04 g.

**Figure 7:**
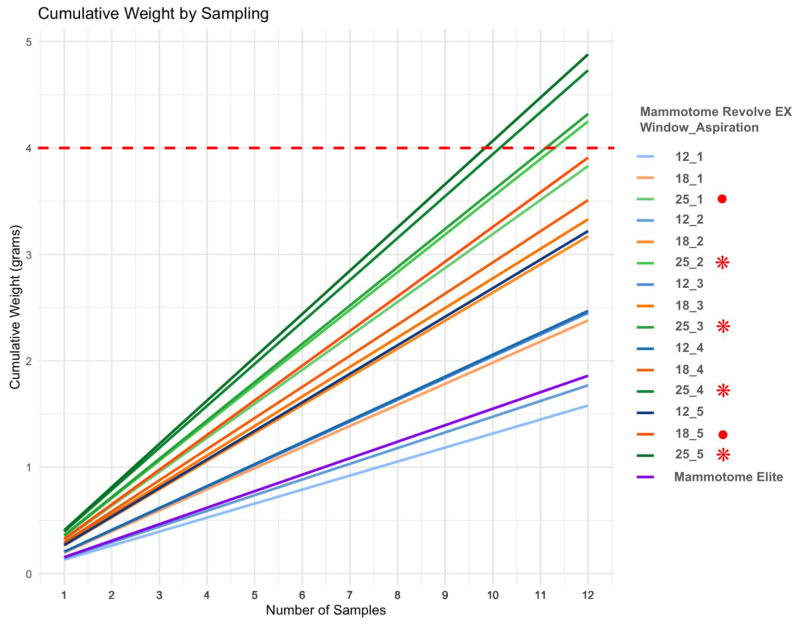
The figure illustrates the cumulative weights of 12 samples, each of equal weight, calculated using the mean weights for each combination of aspiration levels and window lengths with the Mammotome^®^ Revolve EX. It is evident that certain combinations, highlighted by the red asterisks, are able to reach the empirical threshold of 4 g, denoted by the horizontal red line, after 12 samples. Moreover, two additional combinations, highlighted by the red dots, approach the threshold but do not reach it.

Cumulatively, as shown in Figure 7, only four combinations with the 8 G Mammotome^®^ Revolve EX achieved a total weight of 4 g after 12 samples. Notably, all combinations involving the 25 mm window length, except for the first aspiration level, reached this threshold. Additionally, two combinations—the 25 mm window length at aspiration level 1 and the 18 mm window length at aspiration level 5—approached the 4 g threshold but fell slightly short, followed by aspiration level 4 with the 18 mm window length.

Table 2 presents the estimated number of samplings required for each combination of aspiration level and window length and their confidence intervals.

Among the combinations, the smallest number of samples needed to achieve 4 g was observed for the combination of the 25 mm window length and aspiration level 5, requiring only 10 samples (from 8 to 12 samples). This is followed by the 25 mm window length and aspiration level 4, which requires, on average, 10 samples as well (from 8 to 14 samples). On the other hand, combinations with smaller window lengths and lower aspiration levels, such as the 12 mm window length and aspiration level 1, require the most samples, with 30 samples needed to reach 4 g. The Mammotome Elite, in its fixed default configuration, requires around 26 samples to reach 4 g, with a confidence interval ranging from 18 to 47 samples.

The average fragment lengths demonstrated a progressive increase with higher aspiration levels and longer needle windows. These lengths ranged from 13.08 ± 2.15 mm for the 12 mm window length at aspiration level 1 to 35.42 ± 5.71 mm for the 25 mm window length at aspiration level 5. The standard deviations indicated increasing variability as the needle length increased, with the highest variability observed at aspiration level 5 for the 25 mm window length. This trend underscores the influence of both needle length and aspiration intensity on fragment dimensions, particularly for the longest needles.

## 4. Discussion

VAE has gained increasing popularity for the management of breast lesions. Initially utilized for benign lesions [5] and later expanded to B3 and high-risk lesions [18], VAE has recently shown promising potential in the excision of small tumors [11,12]. As applications continue to expand, a thorough understanding of the technical features of the tools available in clinical practice is important for optimizing their potential and enhancing their clinical impact. This knowledge is particularly relevant when addressing the technical challenges associated with VAE procedures. Proficiency in handling the devices is especially valuable when treating patients with small breasts or targeting lesions in anatomically challenging areas, such as near the pectoralis minor muscle. In these cases, a versatile and user-friendly device can significantly facilitate the procedure.

Currently, the Mammotome^®^ Elite serves as one of the standard and well-known VAE devices in clinical practice [19]. This minimally invasive biopsy tool has been used since 2016 in clinical practice. It is portable, easy to handle, and does not require connection to an external power supply. However, it has certain limitations, offering only a single sampling window and one fixed aspiration power level, which restricts its versatility.

In contrast, the Mammotome^®^ Revolve EX is a newer device launched commercially in 2022 and designed to enhance the efficiency and precision of vacuum-assisted excision procedures, providing enhanced flexibility by offering three sampling window lengths (12 mm, 18 mm, and 25 mm) and five aspiration levels. This adaptability allows clinicians to tailor the device’s configuration to various clinical scenarios. To date, however, no studies in the literature have directly compared the performance of the Mammotome^®^ Elite and the Mammotome^®^ Revolve EX across all possible settings.

The adaptability and flexibility of the Mammotome^®^ Revolve EX enable personalized treatment by tailoring the technology to each patient’s specific needs. This approach enhances procedural efficiency by facilitating the selection and application of the most suitable techniques to achieve optimal outcomes.

Our analysis of the samples obtained with the Mammotome^®^ Revolve EX reveals a clear trend: increasing the needle window size and aspiration levels corresponded to a rise in the average weight of the tissue fragments. This underscores the device’s versatility, allowing adjustments based on factors such as breast size, lesion size, and location. For instance, sample weights for the 12 mm window ranged from approximately 0.13 g at lower aspiration levels to 0.27 g at higher levels, while for the 25 mm window, weights ranged from around 0.32 g to 0.41 g.

This wide range of sampling dimensions reflects the device’s ability to provide sufficient flexibility to meet procedural demands, ensuring adequate lesion sampling while minimizing complications. Since the technique is used for excisional purposes, we considered it appropriate to analyze the cumulative performance of VAE in obtaining tissue samples with a volume or weight comparable to those of a diagnostic surgical excision, approximately 4 g [3].

When considering the cumulative excisional capacity, only four specific combinations of window length and aspiration level yielded an estimated total sample weight of 4 g after 12 samples. Most of these involved the 25 mm window length at higher aspiration levels, while the 18 mm window length at the maximum aspiration level also approached this threshold but fell slightly short.

Comparing the Mammotome^®^ Revolve EX to the Mammotome^®^ Elite revealed that the Mammotome^®^ Revolve EX consistently achieved higher average sample weights across all aspiration levels. This superiority was particularly pronounced with the 25 mm and 18 mm windows. However, with the 12 mm window, the Revolve only outperformed the Mammotome^®^ Elite at the highest three aspiration levels. At the two lowest levels, the Mammotome^®^ Revolve EX produced slightly lower sample weights, on average, than the Mammotome^®^ Elite. This finding is significant because it suggests that even with smaller windows, such as the 12 mm window, adequate sampling results can be achieved by increasing the aspiration level. The capability of using smaller windows is crucial for a tailored approach, especially considering the localization of lesions. The smallest window is particularly beneficial for lesions located very close to the skin or the pectoral muscle, where the safety margin is reduced. Using the smaller window allows the operator to feel more confident, enabling an optimal procedure. However, as demonstrated by our test, the smaller window can be paired with a higher level of suction to achieve good performance and minimize patient discomfort while ensuring an appropriate number of samplings relative to the lesion size and location.

Moreover, our data on the estimated number of samplings required based on various combinations of aspiration levels and window lengths shown in Table 1 highlight the interplay between the Mammotome^®^ Revolve EX settings and sampling efficiency, offering insights into how different configurations impact the overall number of samples needed. By analyzing these combinations, it becomes evident how adjustments in aspiration level and window length can optimize the sampling process for specific procedural needs.

Our findings underscore the Mammotome^®^ Revolve EX’s versatility and promising potential for excisional applications. The device’s ability to deliver excellent sampling results—especially with larger windows and/or higher aspiration levels—offers significant clinical prospects. The Mammotome^®^ Revolve EX, due to its technical characteristics, may prove particularly beneficial for treating lesions that are otherwise difficult to access, such as those located in challenging anatomical regions.

This study has several limitations. First, both needles were used for numerous samplings, and the high number of samples collected—particularly with the Mammotome^®^ Revolve EX—may have caused slight wear on the needle, potentially impacting performance during the later samples. Additionally, for measuring sample dimensions, chicken cores were laid flat on a surface. While this process was carried out as consistently as possible, minor variations were unavoidable due to differences in the density of the cores themselves. Another source of bias stems from using different chickens for the samples and, within the same chicken, from different areas of the chicken. These differences may lead to slight variability in tissue composition, such as the proportions of connective and muscular tissue. Overall, these limitations may have contributed to the significant variability in measurements, as reflected by the substantial standard deviations ranging from ±0.026 to ±0.068. This highlights notable inconsistencies that could impact the reliability and comparability of the results in the experimental setting and should be carefully considered in clinical applications.

## 5. Conclusions

In conclusion, considering the importance of VAE and minimally invasive treatments in breast cancer management, as well as the future prospects of these approaches, it is essential to have an in-depth understanding of the available technologies and their specific features. Moreover, a thorough understanding of the technical capabilities is crucial in pre-procedural planning to determine the optimal starting conditions and is fundamental during the procedure for making necessary adjustments, ensuring precision and adaptability for the best clinical outcomes. This study provides valuable evidence that VAE demonstrates excellent excisional capabilities, making it a highly effective tool in the management of B3 lesions and small breast tumors. Furthermore, its versatility—enabled by adjustable window sizes and aspiration levels—allows for the collection of samples with varying dimensions and weight, enabling optimal adaptation of the procedure to the lesion’s characteristics and location and patient comfort, all while maintaining robust excisional capacity.

## Figures and Tables

**Table 2:** Estimated number of samplings required for each combination of aspiration level and window length, along with their confidence intervals, to reach the 4 g threshold, in ascending order of the required number of samples.

Combinations	Mean_Weight	Dev_Std	Mean_N_Samples	IC_Lower	IC_Upper
e_5_25_p	0.41	0.04	9.84	9.82	9.86
e_4_25_p	0.39	0.05	10.15	10.12	10.17
e_3_25_p	0.37	0.08	10.91	10.87	10.95
e_2_25_p	0.35	0.05	11.29	11.27	11.32
e_5_18_p	0.33	0.05	12.28	12.25	12.3
e_1_25_p	0.32	0.03	12.53	12.52	12.55
e_4_18_p	0.29	0.06	13.68	13.65	13.7
e_3_18_p	0.28	0.06	14.41	14.39	14.44
e_5_12_p	0.27	0.05	14.91	14.88	14.93
e_2_18_p	0.26	0.05	15.14	15.12	15.17
e_3_12_p	0.2	0.05	19.59	19.57	19.61
e_4_12_p	0.2	0.03	19.67	19.66	19.69
e_1_18_p	0.2	0.03	20.17	20.16	20.18
Elite	0.16	0.04	25.81	25.79	25.82
e_2_12_p	0.15	0.03	27.12	27.1	27.14
e_1_12_p	0.13	0.03	30.38	30.37	30.39

## Data Availability

The original contributions presented in this study are included in the article. Further inquiries can be directed to the corresponding author.
